# Impact of underweight on 3-year all-cause mortality in patients with acute severe hypertension: a retrospective cohort study

**DOI:** 10.1038/s41598-022-08892-9

**Published:** 2022-03-21

**Authors:** Hyun-Jin Kim, Byung Sik Kim, Jun Hyeok Lee, Jeong-Hun Shin

**Affiliations:** 1 Division of Cardiology, Department of Internal Medicine, Hanyang University College of Medicine, Hanyang University Guri Hospital, 153 Gyeongchun-ro, Guri, Gyeonggi-do 11923 Republic of Korea; 2 Department of Biostatistics, Yonsei University Wonju College of Medicine, Wonju, Republic of Korea

**Keywords:** Cardiology, Diseases, Weight management

## Abstract

Body mass index (BMI) is used to measure body fat. We investigated the association between BMI and long-term clinical outcomes in patients with acute severe hypertension who visited the emergency department (ED). Cross-sectional study data were obtained from a single regional emergency medical center, including patients with elevated initial systolic blood pressure ≥ 180 mmHg or diastolic blood pressure ≥ 100 mmHg. The patients were classified into five groups according to BMI level (underweight, normal, overweight, obese class I, II and III). Among 4867 patients who presented with acute severe hypertension at the ED, 935 (19.21%) died within 3-years. In particular, 140 (44.59%) patients in the underweight group died from any cause, which was the highest among the five groups, and there was a reverse J-shaped association between BMI and 3-year all-cause mortality. Underweight patients had a significantly increased risk of all-cause mortality by 1.55-fold during the 3-year follow-up. Rather, obesity was associated with a reduction in the 3-year all-cause mortality. Comorbidities, including chronic kidney disease and acute hypertension-mediated organ damage, were independent predictors of all-cause mortality in patients who were not underweight. Underweight contributes to worsening long-term clinical outcomes in patients with acute severe hypertension. Clinicians should consider BMI as one of the physical examination parameters in patients with acute severe hypertension, and management including lifestyle modifications such as diet control and exercise should be undertaken considering BMI and comorbidities.

## Introduction

Hypertension is a well-known risk factor for cardiovascular disease (CVD) and all-cause mortality worldwide^1^. Effective lifestyle modification and anti-hypertensive drug treatment strategies can reduce blood pressure (BP) in hypertensive patients^2^. Nevertheless, BP control rates are still poor worldwide and are not satisfactory in Korea or across Europe^3^. In particular, acute and severe BP elevations due to uncontrolled hypertension are serious problems commonly encountered in patients visiting the emergency department (ED). According to our previous study, patients with acute severe hypertension, which was defined as systolic blood pressure (SBP) ≥ 180 mmHg or diastolic blood pressure (DBP) ≥ 100 mmHg, accounted for 59 out of 1000 ED visits at a single center and showed poor clinical outcomes, with a 1-year mortality rate of 8.8% and a 3-year mortality rate of 13.9%^4^.

Body mass index (BMI) is one of the markers of obesity and body fat^5^, which has a strong association with all-cause mortality. Most studies demonstrated a J- or U-shaped association with the lowest mortality in people with an optimal BMI (20–25 kg/m^2^) range^6–9^. However, the obesity paradox implies that overweight and mildly obese groups have a better prognosis for all-cause mortality^10^. Low BMI (< 18.5 kg/m^2^) may be a risk factor for CVD and all-cause mortality, which may be attributed to aging, sarcopenia, and poor nutritional status in the underweight population^11^. Although underweight and obesity are important clinical characteristics of patients with CVD, few studies have attempted to demonstrate the effect of BMI on all-cause death or cardiovascular outcomes in patients with acute severe hypertension.

In this study, we investigated the association of BMI level with all-cause mortality during long-term follow-up in patients with acute severe hypertension who visited the ED.

## Method

### Study population and data collection

**Figure 1:**
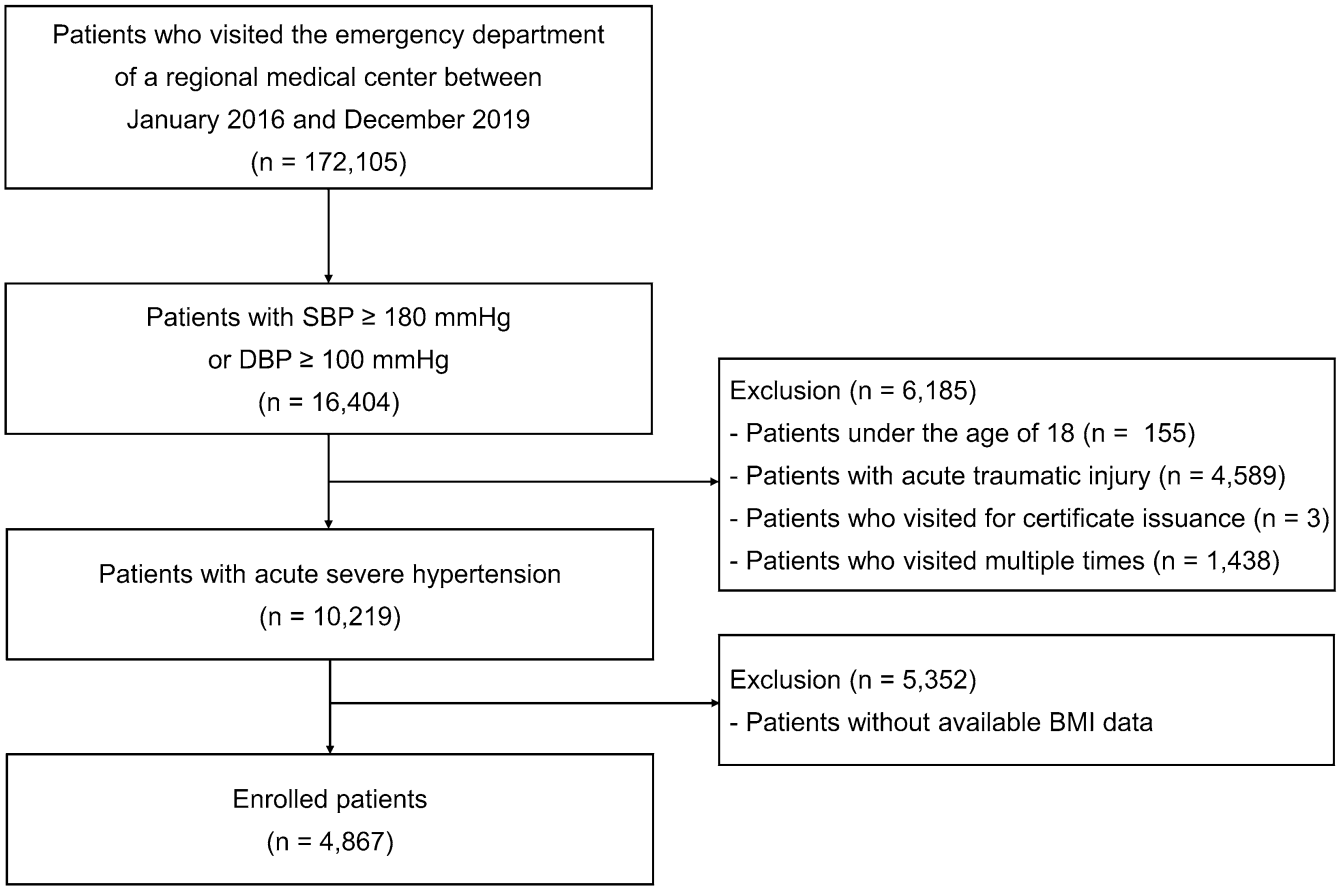
Study population.

The data for this retrospective cohort study were obtained from a single regional emergency medical center: Hanyang University Guri Hospital, Guri, Gyeonggi-do, Korea. The study design and primary results were previously published^4^. The medical records of 172,105 patients who visited the ED between January 2016 and December 2019 were reviewed; 16,404 patients who had elevated initial SBP ≥ 180 mmHg or DBP ≥ 100 mmHg were enrolled in this study (Fig. 1). Of these, patients aged < 18 years who presented with acute trauma, certificates, or who visited the ED multiple times were excluded from this study. Among these 10,219 patients with acute severe hypertension, 4867 had available data regarding BMI. The National Emergency Department Information System (NEDIS) data were used to identify eligible patients who visited the ED. The system collects data, including initial vital signs and baseline clinical characteristics. This information on all patients visiting the ED is automatically transmitted from each hospital to a central government server called NEDIS. The patients were classified into five groups according to BMI level as follows; underweight group whose BMI < 18.5 kg/m^2^, normal group whose BMI between 18.5 kg/m^2^ and 22.9 kg/m^2^, overweight group whose BMI kg/m^2^ between 23 and 24.9 kg/m^2^, obese class I group whose BMI between 25 kg/m^2^ and 29.9 kg/m^2^, obese class II + III group whose BMI ≥ 30 kg/m^2^. The study was conducted in accordance with the Declaration of Helsinki, and was reviewed and approved by the Institutional Review Board of Hanyang University Guri hospital (GURI 2020-01-028). The institutional review board of Hanyang University Guri hospital waived the requirement for written informed consent.

### Data collection

Data were collected using electronic medical records by experienced data collectors under principal investigator supervision. The following demographic and clinical characteristics were extracted: age, sex, initial BP at the ED, and traditional cardiovascular risk factors, including a history of hypertension, diabetes, dyslipidemia, chronic kidney disease (CKD), end-stage renal disease, smoking and alcohol consumption status. History of heart failure, ischemic stroke, hemorrhagic stroke, coronary artery disease, and peripheral artery disease was also extracted.

The following laboratory data were extracted: estimated glomerular filtration rate (eGFR) (mL/min/1.73 m^2^), B-type natriuretic peptide (BNP), and proteinuria in a dipstick urinalysis. In addition, diagnostic test findings to determine the presence of acute hypertension-mediated organ damage (HMOD) were obtained.

The primary outcome was the incidence of all-cause mortality during the 3-year follow-up (until March 15, 2021) according to the BMI level. The secondary outcomes were all-cause mortality within 1-month, 3-months and 1-year follow-up, hospital admission, and death at the ED according to BMI level. The incidence of mortality and its timing were extracted from the National Health Insurance Service in South Korea, and other outcome data were extracted from local medical records.

### Definition

Obesity and overweight were defined according to the WHO guidelines for the Asia Pacific region and the Korean Society for the Study of Obesity Guideline for the management of obesity^12^. Acute HMOD was defined as hypertensive encephalopathy, cerebral infarction, intracerebral hemorrhage, retinopathy, acute heart failure, acute coronary syndrome (ACS), acute renal failure, and aortic dissection^13^. Hypertensive encephalopathy was defined as a severe increase in BP associated with other unexplained lethargy, seizures, cortical blindness, and coma^13^. Cerebral infarction and intracerebral hemorrhage were defined on the basis of neurological symptoms and brain imaging at the ED. Fundus examination confirmed retinopathy based on the presence of flame-like hemorrhages, cotton wool spots, or papilledema. Proteinuria was defined as a dipstick urinalysis result of ≥ 1 +^14^. BP was measured in the ED above the brachial artery using an automated BP machine, Spot Vital Signs LXi (Welch Allyn, Skaneateles Falls, NY, USA).

### Statistical analysis

All categorical data are presented as numbers and percentages, while statistics for continuous variables are presented as means and standard deviations. The Cochran–Mantel–Haenszel test was used to extract the trend of clinical outcomes, including mortality, hospital admission, and death at the ED, and categorical data of baseline characteristics according to BMI level. Linear regression was used to show the distribution of the means and the trend of continuous variables of baseline characteristics according to BMI. Kaplan–Meier survival analyses and log-rank tests were used to compare the cumulative survival probability according to BMI level. The restricted cubic spline curve showed a continuous adjusted association between BMI and the risk of all-cause mortality in patients with acute severe hypertension. Hazard ratio (HR) and respective 95% confidence intervals (CIs) for 3-year all-cause mortality in the underweight, overweight, and obese groups compared with the normal BMI group were calculated using multivariable Cox proportional hazards regression analyses. We investigated the association between BMI level and 3-year all-cause mortality, with and without adjustment for the selected confounders. Three models were used. In Model 1, age and sex were considered as possible confounders. Model 2 included factors which were thought to be clinically relevant (age, sex, and social history) and medical history with *p* < 0.05 at baseline, as follows: age, sex, smoking, alcohol consumption, and medical history of hypertension, diabetes mellitus, dyslipidemia, ischemic stroke, hemorrhagic stroke, chronic kidney disease, and end-stage renal disease. Model 3 included variables with *p* < 0.2 in the univariable Cox proportional hazards regression analyses, as follows: age, sex, smoking, alcohol consumption, and medical history of hypertension, diabetes mellitus, dyslipidemia, ischemic stroke, hemorrhagic stroke, coronary artery disease, heart failure, chronic kidney disease, and end-stage renal disease. To evaluate the risk factors for 3-year all-cause mortality of each BMI category, we performed the multivariable Cox proportional hazards regression analyses using the variables with *p* < 0.2 in the univariable analysis of each group. Additionally, we performed a subgroup analysis with the multivariable Cox proportional hazards regression analyses according to the age group (< 50, 50–59, 60–69, and ≥ 70 years), presence or absence of a history of diabetes mellitus, and patients without chronic disease, including heart failure, ischemic stroke, hemorrhagic stroke, and end-stage renal disease. Among the candidate variables, social history and medical history were missing for some patients. History of alcohol consumption and cigarette smoking was missing for 62 (1.3%) and 65 patients (1.3%), respectively, and end-stage renal disease, which had the highest missing data in the medical history, was missing for 31 patients (0.6%). We conducted the Cox proportional hazard regression with complete-case analysis, which involves restricting the analysis to individuals with no missing data. We tested the proportional hazards assumption for each variable in the multivariable models using the supremum test. All variables in the models were found to satisfy the proportional hazards assumption. All tests were two-tailed, and statistical significance was set at *p* < 0.05. All analyses were performed using the Statistical Analysis Software (version 9.4; SAS Institute, Cary, NC, USA).

## Results

### Baseline characteristics

Among 4867 patients with acute severe hypertension at the ED, 314 (6.45%) patients in the underweight group, 1528 (31.40%) in the normal group, 1026 (21.08%) in the overweight group, 1530 (31.44%) patients in the obese class I group, and 469 (9.64%) patients in the obese class II + III group were analyzed in the present study. The baseline characteristics of the patients are presented in Table 1. The mean age of the total study population was 61.85 ± 16.45 years, and half were male (53.61%). The patients in the underweight group were the oldest, and most frequently female among the five groups. Patients in the obese class II + III group had the highest prevalence of current smokers and alcohol drinkers, and had the highest incidence of traditional cardiovascular risk factors, such as hypertension, diabetes mellitus, and dyslipidemia among the five groups. On the contrary, ischemic stroke and hemorrhagic stroke were the most common in the underweight group. In laboratory findings, the lower the BMI, the higher the BNP, and the higher the prevalence of proteinuria. In the diagnostic test findings to evaluate the presence of HMOD, cardiomegaly was significantly more common as BMI increased. There was no difference among the groups in the frequency of taking antihypertensive medication among patients with hypertension. The patterns of acute HMOD according to BMI are shown in Table 1. Acute coronary syndrome and hypertensive retinopathy showed a tendency to increase significantly as BMI increased, while acute heart failure showed a tendency to increase significantly as BMI decreased. There was no significant difference according to BMI in the other acute HMODs.

**Table 1:** Baseline characteristics.

	Overall patients (n = 4867)	Body mass index (kg/m^2^)					*p* value for trend
		Underweight (BMI < 18.5 kg/m^2^) (n = 314)	Normal (18.5 ≤ BMI ≤ 22.9 kg/m^2^) (n = 1528)	Overweight (23.0 ≤ BMI ≤ 24.9 kg/m^2^) (n = 1026)	Obese class I (25.0 ≤ BMI ≤ 29.9 kg/m^2^) (n = 1530)	Obese class II + III (BMI ≥ 30.0 kg/m^2^) (n = 469)	
**Mean age, years (SD)**	61.85 (16.45)	68.44 (17.91)	64.68 (16.64)	63.62 (14.91)	59.63 (14.97)	51.52 (17.11)	< 0.001
**Male sex, N (%)**	2609 (53.61)	133 (42.36)	750 (49.08)	575 (56.04)	897 (58.63)	254 (54.16)	< 0.001
**Medical history, N (%)**							
Hypertension	2696 (55.50)	159 (50.96)	805 (52.79)	590 (57.50)	877 (57.47)	265 (56.50)	0.005
Diabetes mellitus	1372 (28.28)	74 (23.72)	418 (27.45)	284 (27.76)	441 (28.88)	155 (33.19)	0.006
Dyslipidemia	628 (12.97)	12 (3.85)	162 (10.67)	160 (15.64)	222 (14.58)	72 (15.48)	< 0.001
Ischemic stroke	445 (9.19)	44 (14.10)	158 (10.39)	89 (8.69)	129 (8.48)	25 (5.38)	< 0.001
Hemorrhagic stroke	182 (3.76)	18 (5.77)	68 (4.48)	47 (4.59)	33 (2.17)	16 (3.44)	< 0.001
Coronary artery disease^a^	511 (10.55)	28 (8.97)	154 (10.13)	110 (10.76)	181 (11.87)	38 (8.15)	0.561
Peripheral artery disease	59 (1.22)	1 (0.32)	23 (1.52)	11 (1.08)	18 (1.18)	6 (1.29)	0.888
Heart failure	260 (5.37)	22 (7.05)	90 (5.93)	52 (5.08)	70 (4.60)	26 (5.59)	0.103
Chronic kidney disease	429 (8.86)	38 (12.18)	161 (10.60)	94 (9.19)	102 (6.71)	34 (7.28)	< 0.001
End-stage renal disease	193 (3.99)	27 (8.65)	82 (5.41)	31 (3.03)	38 (2.50)	15 (3.22)	< 0.001
**Social history, N (%)**							
Cigarette smoking	1465 (30.51)	87 (28.16)	428 (28.42)	295 (29.06)	479 (31.72)	176 (38.10)	< 0.001
Alcohol consumption	1761 (36.65)	80 (25.89)	498 (33.02)	375 (36.95)	615 (40.76)	193 (41.59)	< 0.001
**Initial blood pressure, mean (SD)**							
SBP, mmHg	181.44 (25.82)	181.20 (27.71)	180.01 (25.88)	183.30 (25.54)	181.86 (25.48)	180.82 (25.80)	0.137
DBP, mmHg	105.91 (15.10)	105.65 (16.69)	104.50 (25.88)	105.48 (14.56)	106.78 (14.96)	108.78 (15.39)	< 0.001
**Laboratory tests**							
Mean eGFR, mL/min/1.73 m^2^ (SD)	79.21 (31.06)	73.33 (35.18)	76.48 (32.51)	78.57 (30.27)	81.43 (28.35)	86.65 (31.54)	< 0.001
BNP, pg/mL (SD)	422.04 (844.86)	711.91 (1138.01)	547.04 (976.20)	347.01 (725.86)	311.71 (694.03)	268.86 (617.08)	< 0.001
Proteinuria^b^, N (%)	1041 (32.26)	88 (39.82)	328 (32.44)	209 (30.74)	326 (31.99)	90 (30.41)	0.102
**Chest X-ray done, N (%)**	4314 (88.64)	286 (91.08)	1350 (88.35)	916 (89.28)	1363 (89.08)	399 (85.07)	0.132
Cardiomegaly, N (%)	607 (13.80)	33 (11.42)	166 (12.06)	139 (14.85)	201 (14.85)	68 (16.31)	0.007
**Brain imaging done, N (%)**	1660 (34.11)	100 (31.85)	550 (35.99)	370 (36.06)	512 (33.46)	128 (27.29)	0.017
Abnormal finding, N (%)	741 (35.87)	50 (40.65)	247 (36.43)	200 (44.25)	197 (31.07)	47 (26.26)	< 0.001
**Chest and abdomen CT done, N (%)**	611 (12.55)	62 (19.75)	205 (13.42)	121 (11.79)	165 (10.78)	58 (12.37)	< 0.001
**Echocardiography done, N (%)**	181 (3.72)	8 (2.55)	48 (3.14)	49 (4.78)	58 (3.79)	18 (3.84)	0.216
**Fundoscopy done, N (%)**	80 (1.64)	2 (0.64)	13 (0.85)	19 (1.85)	36 (2.35)	10 (2.13)	0.007
Abnormal finding, N (%)	29 (5.23)	1 (3.57)	7 (3.98)	4 (3.28)	13 (7.51)	4 (7.14)	0.112
**Current antihypertensive medication, N (%)**	584 (25.80)	38 (28.79)	154 (22.61)	124 (24.60)	207 (28.75)	61 (26.87)	0.085
**Acute HMOD, N (%)**							
Acute ischemic stroke	618 (12.71)	26 (8.28)	206 (13.48)	170 (16.57)	181 (11.86)	35 (7.46)	0.078
Acute coronary syndrome	282 (5.80)	8 (2.56)	70 (4.58)	62 (6.06)	118 (7.71)	24 (5.13)	< 0.001
Acute heart failure	438 (9.01)	39 (12.50)	154 (10.08)	88 (8.59)	121 (7.91)	36 (7.69)	0.003
Intracerebral hemorrhage	228 (4.69)	23 (7.32)	71 (4.65)	54 (5.26)	60 (3.92)	20 (4.26)	0.057
Acute renal failure	279 (5.74)	22 (7.03)	102 (6.68)	53 (5.17)	71 (4.64)	31 (6.61)	0.090
Subarachnoid hemorrhage	106 (2.18)	6 (1.91)	32 (2.10)	27 (2.63)	35 (2.29)	6 (1.28)	0.740
Hypertensive encephalopathy	38 (0.78)	1 (0.32)	16 (1.05)	7 (0.68)	13 (0.85)	1 (0.21)	0.433
Hypertensive retinopathy	32 (0.66)	0 (0.00)	6 (0.39)	7 (0.68)	11 (0.72)	8 (1.71)	0.003
Aortic dissection	23 (0.47)	1 (0.32)	8 (0.52)	4 (0.39)	6 (0.39)	4 (0.85)	0.639

Values for continuous variables are presented as means (SD), and categorical variables are presented as numbers (percentage).

BMI, body mass index; SD, standard deviation; SBP, systolic blood pressure; DBP, diastolic blood pressure; eGFR, estimated glomerular filtration rate; BNP, B-type natriuretic peptide; CT, computed tomography; HMOD, hypertension-mediated organ damage.

^a^Coronary artery disease was defined as a composite history of coronary artery disease, percutaneous coronary intervention, or coronary artery bypass graft surgery.

^b^Proteinuria was defined as a dipstick urinalysis result ≥ 1 +.

### All-cause mortality according to BMI level in patients with acute severe hypertension

**Figure 2:**
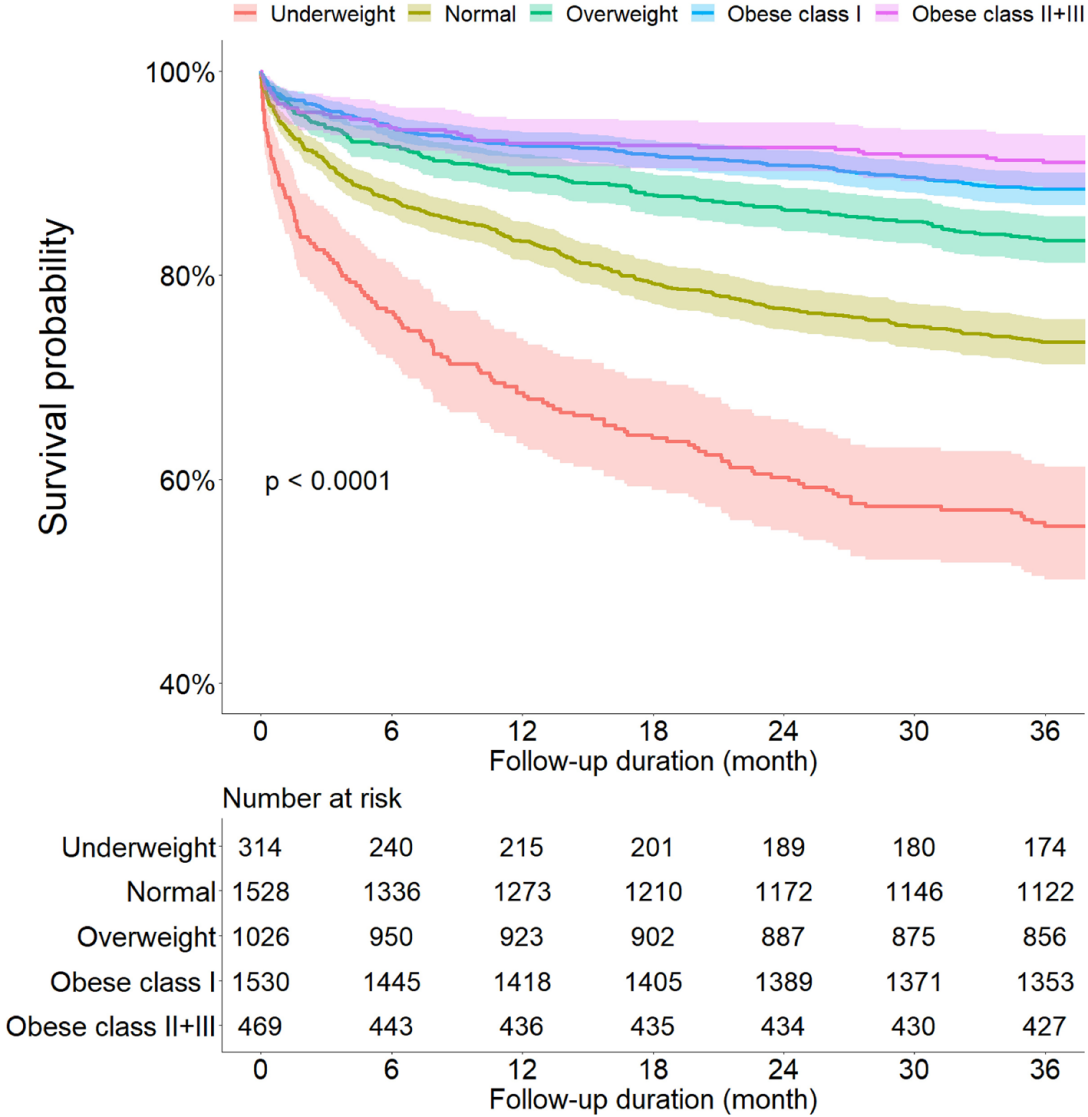
All-cause death free survival rates according to BMI level analysis by Kaplan–Meier plot with 95% confidence intervals. BMI, body mass index.

Of all the patients with data on mortality (n = 4867), a total of 935 (19.21%) patients died within 3 years (Table 2). The trend of primary outcome increased significantly as BMI decreased. In the underweight group, 140 (44.59%) patients died from any cause during the 3-year follow-up period. In the obese II + III group, 42 (8.96%) patients, lowest among the five groups, died from any cause during the 3-year follow-up. As a secondary outcome, the trend of all-cause mortality rates increased significantly as BMI decreased at 1-month, 3-month, and 1-year follow-ups. Indeed, 3461 (71.11%) patients were admitted, and one patient (0.02%) died in the ED. Underweight patients showed the highest admission rates among the five groups. Based on the BMI level, cumulative death-free survival probability was analyzed, and the results are shown in Fig. 2. Among the five groups according to BMI level, in unadjusted comparisons, the cumulative survival probability was significantly lower in the underweight group. The five groups according to BMI had significantly different probabilities of all-cause mortality (log-rank *p* < 0.001).

**Table 2:** Clinical outcome.

	Overall patients (n = 4867)	Body mass index (kg/m^2^)					*p* value for trend
		Underweight (BMI < 18.5 kg/m^2^) (n = 314)	Normal (18.5 ≤ BMI ≤ 22.9 kg/m^2^) (n = 1528)	Overweight (23.0 ≤ BMI ≤ 24.9 kg/m^2^) (n = 1026)	Obese class I (25.0 ≤ BMI ≤ 29.9 kg/m^2^) (n = 1530)	Obese class II + III (BMI ≥ 30.0 kg/m^2^) (n = 469)	
Admission	3461 (71.11)	248 (78.98)	1120 (73.30)	732 (71.35)	1047 (68.43)	314 (66.95)	< 0.001
Death in the emergency department	1 (0.02)	0 (0.00)	1 (0.07)	0 (0.00)	0 (0.00)	0 (0.00)	0.345
**Mortality**							
1-month mortality	196 (4.03)	36 (11.46)	79 (5.17)	28 (2.73)	38 (2.48)	15 (3.20)	< 0.001
3-month mortality	326 (6.70)	56 (17.83)	135 (8.84)	57 (5.56)	58 (3.79)	20 (4.26)	< 0.001
1-year mortality	602 (12.37)	99 (31.53)	255 (16.69)	103 (10.04)	112 (7.32)	33 (7.04)	< 0.001
3-year mortality	935 (19.21)	140 (44.59)	406 (26.57)	170 (16.57)	177 (11.57)	42 (8.96)	< 0.001

Values are given as numbers (percentage).

### Association between BMI and all-cause mortality

**Figure 3:**
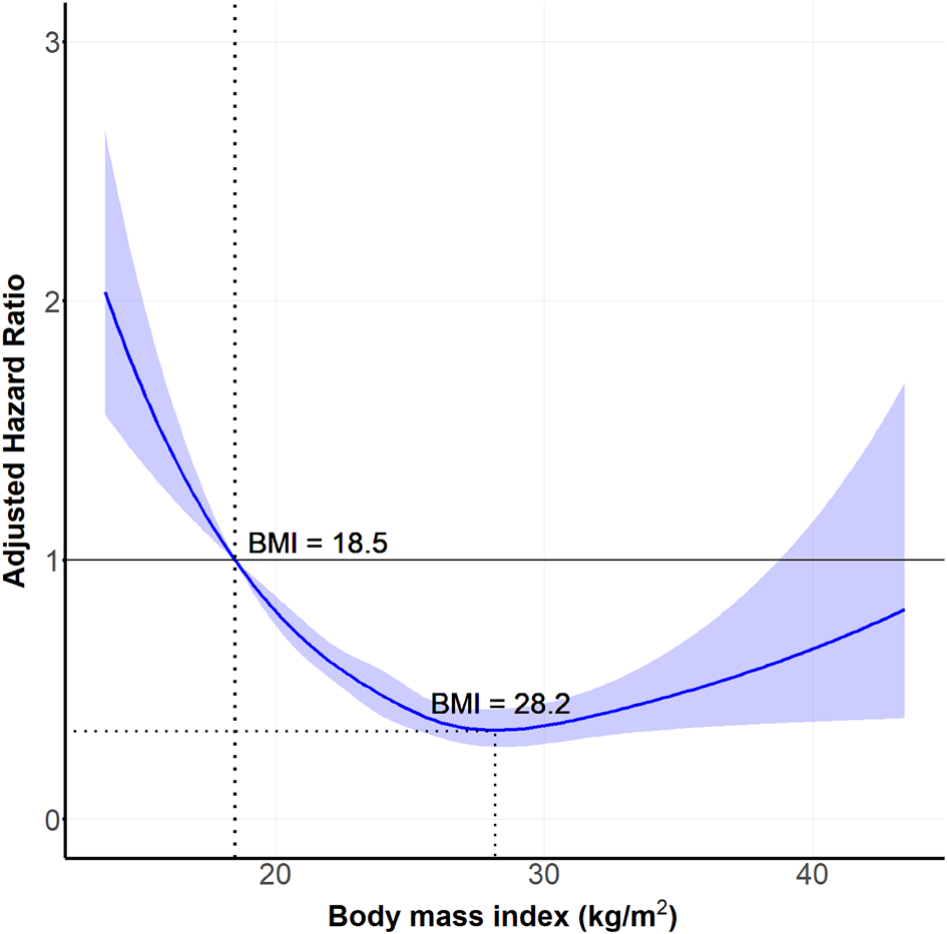
Continuous association between BMI and all-cause mortality in acute severe HTN patients. Restricted cubic splines showing a continuous adjusted association between BMI and risk of all-cause mortality in patients with HTN crisis. The shaded area shows the 95% confidence interval for the estimated hazard ratio point associated with different BMI levels after adjusting for age, sex, hypertension, diabetes mellitus, dyslipidemia, ischemic stroke, hemorrhagic stroke, chronic kidney disease, end-stage renal disease, smoking, and alcohol consumption. (Reference BMI :18.5 kg/m^2^). BMI, body mass index; CI, confidence interval; HTN, hypertension.

Compared with patients with a normal BMI, those with a higher BMI were associated with a graded inverse association with mortality (Table 3). The overweight group was associated with a 41% lower risk in all-cause mortality, a 60% lower risk in the obese class I group, and a 69% lower risk in the obese class II + III group. In contrast, the underweight group had a 1.96-fold increased risk in all-cause mortality. Similar findings were observed in the adjusted analysis, which showed a significant, inverse association between BMI and all-cause mortality risk, and the underweight group was significantly associated with a 1.55-fold increase in all-cause mortality. Similar findings were also observed in the continuous analysis using restricted cubic splines with a reverse J-shaped association between BMI and the risk of all-cause mortality (Fig. 3). The nadir for all-cause mortality risk among patients with acute severe hypertension was estimated from model 3 to be 28.2 kg/m^2^. There was no serious violation of the proportional hazard assumption, as the *p* values for all adjusted covariates using the supremum test were greater than 0.05 (Supplementary Table S1).

**Table 3:** Adjusted association between BMI categories and risk of all-cause mortality in patients with acute severe hypertension.

	Adjusted hazard ratios and 95% confidence interval for 3-year all-cause mortality				
	Underweight	Normal	Overweight	Obese class I	Obese class II + III
Unadjusted	1.96 (1.59–2.34)	Ref	0.59 (0.49–0.70)	0.40 (0.33–0.47)	0.31 (0.22–0.43)
Model 1^a^	1.62 (1.33–1.96)	Ref	0.62 (0.52–0.75)	0.53 (0.44–0.63)	0.60 (0.44–0.83)
Model 2^b^	1.55 (1.27–1.89)	Ref	0.63 (0.53–0.76)	0.53 (0.44–0.63)	0.60 (0.43–0.83)
Model 3^c^	1.56 (1.28–1.90)	Ref	0.64 (0.53–0.76)	0.53 (0.44–0.63)	0.58 (0.42–0.81)

The body-mass index was < 18.5 in underweight group, 18.5 to 22.9 in normal group, 23.0 to 24.9 in overweight subjects, 25.0 to 29.9 in obese class I group, and ≥ 30.0 in obese class II + III group. BMI, body mass index. Values are presented as the hazard ratio (95% confidence interval).

^a^Adjusted for age and sex.

^b^Adjusted for age, sex, hypertension, diabetes mellitus, dyslipidemia, ischemic stroke, hemorrhagic stroke, chronic kidney disease, end-stage renal disease, smoking, and alcohol.

^c^Adjusted for age, sex, hypertension, diabetes mellitus, dyslipidemia, ischemic stroke, hemorrhagic stroke, coronary artery disease, heart failure, chronic kidney disease, end-stage renal disease, smoking, and alcohol, which were variables with *p* value < 0.2 in the univariate analysis.

### Predictors for 3-year all-cause mortality of each BMI group

Table 4 shows the Cox proportional hazards regression analysis according to BMI level after adjusting for confounding factors. (Supplementary Table S2 shows the univariate analysis to evaluate the predictors of 3-year all-cause mortality in each BMI group.) Old age was independently associated with 3-year mortality in all groups stratified by BMI. Except for age, the predictors of long-term mortality were slightly different for each BMI level. Male sex and diabetes mellitus (HR 1.97, 95% CI 1.38–2.83, *p* < 0.001; HR 1.54, 95% CI 1.04–2.21, *p* = 0.018) were significant independent predictors of all-cause mortality in the underweight group. In the normal group, male sex, CKD, smoking, and the presence of acute HMOD at the ED were independent predictors. In the overweight group, ischemic stroke, CKD, and the presence of acute HMOD at the ED were independent predictors. Dyslipidemia, CKD, and the presence of acute HMOD at the ED were independent predictors of 3-year mortality in the obese class I group, whereas in the obese class II + III group, CKD and the presence of acute HMOD at the ED were significant predictors during the long-term follow-up.

**Table 4:** Predictors for 3-year all-cause mortality of each BMI group.

Variables	Underweight		Normal		Overweight		Obese class I		Obese class II + III	
	HR (95% CI)	*p* value	HR (95% CI)	*p* value	HR (95% CI)	*p* value	HR (95% CI)	*p* value	HR (95% CI)	*p* value
Age, years	1.05 (1.03–1.06)	< 0.001	1.06 (1.05–1.07)	< 0.001	1.06 (1.04–1.07)	< 0.001	1.06 (1.05–1.08)	< 0.001	1.06 (1.03–1.09)	< 0.001
Male sex	1.97 (1.38–2.83)	< 0.001	1.51 (1.21–1.88)	< 0.001	1.08 (0.77–1.52)	0.669	–	–	0.74 (0.33–1.64)	0.450
Hypertension	0.93 (0.63–1.36)	0.694	0.99 (0.79–1.24)	0.920	1.07 (0.73–1.58)	0.715	0.90 (0.62–1.31)	0.586	0.50 (0.22–1.15)	0.103
Diabetes mellitus	1.51 (1.04–2.21)	0.032	1.13 (0.90–1.41)	0.302	1.06 (0.75–1.50)	0.756	1.40 (1.02–1.94)	0.039	1.04 (0.52–2.05)	0.921
Dyslipidemia	–	–	–	–	–	–	0.48 (0.28–0.82)	0.008	0.95 (0.44–2.08)	0.906
Ischemic stroke	1.19 (0.75–1.88)	0.468	1.12 (0.86–1.46)	0.405	1.73 (1.16–2.59)	0.008	1.35 (0.90–2.03)	0.148	1.07 (0.38–3.03)	0.898
Hemorrhagic stroke	–	–	1.33 (0.88–2.00)	0.179	1.53 (0.85–2.74)	0.155	–	–	–	–
Coronary artery disease	1.12 (0.65–1.93)	0.690	0.99 (0.73–1.32)	0.925	–	–	0.94 (0.63–1.41)	0.776	0.73 (0.29–1.84)	0.501
Peripheral artery disease	–	–	1.11 (0.58–2.12)	0.747	–	–	–	–		
Heart failure	1.51 (0.87–2.62)	0.144	0.97 (0.68–1.38)	0.854	1.41 (0.90–2.22)	0.776	1.41 (0.90–2.22)	0.137	1.73 (0.74–4.06)	0.208
Chronic kidney disease	1.32 (0.82–2.13)	0.258	1.55 (1.09–2.22)	0.015	2.11 (1.32–3.36)	0.002	2.09 (1.33–3.28)	0.002	3.25 (1.31–8.08)	0.011
End-stage renal disease	–	–	1.27 (0.78–2.05)	0.338	1.05 (0.48–2.28)	0.902	0.81 (0.38–1.72)	0.582	1.95 (0.51–7.51)	0.332
Cigarette smoking	–	–	1.22 (0.92–1.61)	0.174	1.08 (0.68–1.73)	0.750	0.95 (0.61–1.46)	0.808	–	–
Alcohol consumption	1.02 (0.61–1.68)	0.954	0.69 (0.51–0.92)	0.012	0.66 (0.42–1.05)	0.077	0.84 (0.55–1.28)	0.416	1.11 (0.44–2.76)	0.830
Acute HMOD	1.18 (0.82–1.70)	0.368	1.38 (1.13–1.69)	0.002	1.48 (1.08–2.04)	0.015	1.69 (1.24–2.32)	0.001	3.09 (1.57–6.05)	0.001

The body mass index was < 18.5 in the underweight group, 18.5 to 22.9 in the normal group, 23.0 to 24.9 in the overweight subjects, 25.0 to 29.9 in the obese class I group, and ≥ 30.0 in the obese class II + III group.

BMI, body mass index; HR, hazard ratio; CI, confidence interval; HMOD, hypertension-mediated organ damage.

### Subgroup analysis for factors that may affect the association between underweight and all-cause mortality

Multivariable Cox proportional hazards regression analyses according to age groups (< 50, 50–59, 60–69, and ≥ 70 years) (Supplementary Table S3) revealed that underweight was associated with an increased risk of all-cause mortality in all the age groups except in the group under the age of 50 years. The results of stratified analysis according to the presence or absence of diabetes mellitus showed a similar trend to the results of overall patients. In addition, the results of patients without chronic disease, including heart failure, ischemic stroke, hemorrhagic stroke, and end-stage renal disease, showed that underweight was also associated with an increased risk of all-cause mortality.

## Discussion

Based on the results of this study, underweight patients with acute severe hypertension had a significantly increased risk of 3-year all-cause mortality, and obese patients were associated with a reduction in 3-year all-cause mortality. There was a reverse J-shaped association between BMI and all-cause mortality, with the lowest mortality at 28 kg/m^2^. In particular, 44.59% of underweight patients died within 3 years. In addition, underweight patients showed the highest admission rates among the five groups. In the non-underweight groups, including normal, overweight, and obese groups, comorbidities including CKD and the presence of acute HMOD at the ED were important risk factors for 3-year all-cause mortality.

In the overall population, BMI and all-cause mortality are known to have a J-shaped association^6,8^. In Korean data that presented association between mortality and BMI in elderly population over 65 years of age using National Health Insurance System-Senior Database^15^, the risk of all-cause mortality was increased by 2.5-times in the underweight group with BMI of < 18.5 kg/m^2^, and 1.4-times in the group with BMI between 18.5 and 23 kg/m^2^, whereas the overweight and obese group with a BMI > 25 kg/m^2^ did not differ significantly in mortality risk from the reference group (BMI 23–25 kg/m^2^). This pattern was also observed in the association between BMI and death due to CVD. It is important to note that underweight adults with a BMI of less than 18.5, have a higher risk of mortality than adults with a normal BMI. To our knowledge, studies on the association between BMI and mortality in hypertensive patients are rare, and there are no studies specifically targeting patients with acute severe hypertension. Xu et al.^16^ demonstrated a U-shaped association between BMI and mortality in patients with hypertension using the Health Improvement Network primary care research database in the United Kingdom, which showed that the mortality risk was increased in the underweight group and there was no increase in risk in overweight and normal BMI groups, and then increased in the obese group with a BMI of 29 kg/m^2^ or higher. In hypertensive Chinese patients, Wei Yang et al.^17^ suggested an association between BMI and all-cause mortality as a reverse J-shape, and even after adjusting for potential confounding factors, the mortality rate of the underweight group increased 1.56-fold compared to normal, and the optimal BMI was 30–34.9 kg/m^2^. Our study is different from previous studies wherein analysis was conducted on patients who visited the ED with acute severe hypertension (initial SBP ≥ 180 mmHg or DBP ≥ 100 mmHg). Our study demonstrated that the underweight group had a 1.55-fold increase in all-cause mortality compared to the normal BMI group, and the relationship between BMI and mortality showed a reverse J-shaped association rather than a U-shaped or J-shaped association. We performed subgroup analysis to exclude the effects of old age and chronic disease status, including heart failure, ischemic stroke, hemorrhagic stroke, and end-stage renal disease, which were highly associated with underweight, and suggested an association between death and underweight. These results of stratified analysis according age did not change with our main results except for those under the age of 50 years, that is, the effect of underweight on 3-year mortality was predominantly found in elderly. In addition, the overweight and obese groups support the obesity paradox that lowered the risk of mortality in patients with acute severe hypertension. The BMI, which corresponds to the lowest risk of all-cause mortality in patients with acute severe hypertension, was estimated to be 28.0 kg/m^2^ even after correcting possible correction factors. Previous Asian studies have shown that the optimal BMI shifts upward, lowering the mortality rate of the overall population, which is consistent with the findings of our study^17–19^.

Obesity, a modifiable risk factor for CVD, is a determinant of adiposity; however, the proportion of lean mass is important. Body composition varies widely among individuals, even if they have the same BMI, because the composition of fat mass and lean mass is different and can affect health outcomes, including mortality^20^. Loss of lean mass is associated with a hyper-inflammatory status, insulin resistance, and cardiometabolic disorders^21,22^. In addition, a smaller lean mass is associated with worse exercise capacity and cardiorespiratory fitness, which could in turn lead to increased mortality^22,23^. A previous Korean study using the Korean National Health Insurance Service database showed that the risk of CVD increases as the severity of underweight increases^24^. Thus, the clinical significance of underweight with a small lean mass is important. The reduced lean mass leads to a lower cardiorespiratory fitness associated with peripheral muscular and cardiac dysfunction, which may play a crucial role in the pathophysiology of heart failure progression^25,26^. The importance of lean mass is also shown in the pattern of acute HMOD in the underweight group, and they had significantly more heart failure than the overweight and obese groups in our study.

Underweight can increase the mortality in patients with acute severe hypertension, rather than due to comorbidities other than age and diabetes. In contrast, CKD and acute HMOD were independent risk factors of all-cause mortality in patients with acute severe hypertension who were not in the underweight group. Renal function plays an important role in BP regulation, and renal dysfunction is also a predictor of poor cardiovascular outcome and death in hypertensive crisis^27,28^. In addition, HMOD consists of structural and functional changes in the arteries or organs and increases the risk of mortality and cardiovascular events in hypertensive patients^1,29^. Although obesity and overweight lower the mortality rate of patients with acute severe hypertension, active management is required for patients with these predictors.

Acute severe hypertension is associated with adverse outcomes and high utilization of healthcare services. In this context, understanding the prognostic factors of all-cause mortality in patients presenting with acute severe hypertension is pivotal in improving the medical care for hypertensive patients while reducing the healthcare burden. In the setting of the ED, it is not possible to perform complete workup in patients with acute severe hypertension because of the difficulty in evaluating indicators for predicting mortality. As this study indicated that diagnostic examination as well as BMI are predictors of mortality, more aggressive follow-up and treatment are required for underweight patients.

Our study had several limitations. First, this was a retrospective study. Patients who visited the ED were not obligated to measure height and weight; therefore, only 47.6% of all patients with acute severe hypertension were available for BMI data; therefore, the possibility of selection bias cannot be excluded. In addition, there were missing data on variables highly related to BMI, such as alcohol consumption, smoking status, exercise, and drug history. We conducted an analysis on all patients with BMI data (n = 4867), despite missing other data. In particular, the obese I group showed that dyslipidemia reduced the risk of mortality, possibly because the information on whether or not dyslipidemia was based on medical record investigations, and having dyslipidemia may mean that the patient is already taking a lipid-lowering drug. If there were data on drug history, an interpretation would have been possible. These missing data and variables that were poorly measured or unmeasured could act as confounders in our results. A prospective study will be needed to develop a protocol that includes all factors that can affect BMI in the future. Second, this study was a single-center experience, which may not be representative of the entire population. Third, because there was no available data on the cause of death from the National Health Insurance Service, cardiovascular mortality could not be determined in this study. However, the data provided by the National Health Insurance Service on all-cause mortality and the date of death are accurate and reliable. Fourth, since echocardiography, fundoscopy, chest, and abdominal CT were performed only in a small number of patients owing to the ED environment and condition, the interpretation of the results of the analysis, to classify by BMI category, is limited.

In conclusion, although high BMI is known to be an important cardiovascular risk factor, underweight rather than overweight and obesity contributes to a worse long-term clinical outcome in patients with acute severe hypertension. Depending on whether the acute severe hypertensive patients are underweight, overweight, or obese, clinicians should make an appropriate approach and management including lifestyle modifications such as diet control and exercise considering their comorbidities and BMI itself.

## Supplementary Information

Supplementary Information.

## Data Availability

The datasets generated during the current study are available from the corresponding author on reasonable request.
