# Supplementary material for: Impact of underweight on 3-year all-cause mortality in patients with acute severe hypertension: a retrospective cohort study
Source: Sci Rep. 2022 Mar 21;12:4798. doi: 10.1038/s41598-022-08892-9 (PMC8938442; doi:10.1038/s41598-022-08892-9)
Supplement: Supplementary file 1 — Supplementary Information. [file 41598_2022_8892_MOESM1_ESM.pdf]

Supplementary Materials

Supplementary Table S1. Proportional hazard assumption

Proportional hazard assumption*	*p*-value
Age	0.0887
Sex	0.5377
Hypertension	0.0700
Diabetes mellitus	0.0603
Dyslipidemia	0.4740
Ischemic stroke	0.2793
Hemorrhagic stroke	0.5097
Coronary artery disease	0.9737
Peripheral artery disease	0.1673
Heart failure	0.2103
Chronic kidney disease	0.0513
End-stage renal disease	0.1103
Cigarette smoking	0.6683
Alcohol consumption	0.1767
Acute HMOD	0.0510

* proportional hazard assumption using Supremum test

Supplementary Table S2. Univariate analysis for predictors of 3-year all-cause mortality of each BMI group

Variables	Underweight		Normal		Overweight		Obese class I		Obese class II+III	
	HR (95% CI)	*p*-value	HR (95% CI)	*p*-value	HR (95% CI)	*p*-value	HR (95% CI)	*p*-value	HR (95% CI)	*p*-value
Age, years	1.04(1.03-1.06)	<0.001	1.06(1.05-1.07)	<0.001	1.07(1.05-1.08)	<0.001	1.07(1.06-1.09)	<0.001	1.07(1.05-1.09)	<0.001
Male sex	1.50(1.08-2.09)	0.016	1.17(0.96-1.42)	0.122	0.77(0.57-1.04)	0.082	0.86(0.64-1.15)	0.310	0.46(0.24-0.86)	0.015
Hypertension	1.80(1.28-2.54)	<0.001	1.95(1.59-2.40)	<0.001	2.50(1.76-3.55)	<0.001	2.25(1.61-3.16)	<0.001	2.20(1.10-4.37)	0.025
Diabetes mellitus	1.79(1.25-2.55)	0.001	1.61(1.32-1.97)	<0.001	1.78(1.31-2.43)	0.003	2.49(1.86-3.35)	<0.001	2.29(1.25-4.19)	0.007
Dyslipidemia	0.74(0.28-2.01)	0.560	0.90(0.65-1.24)	0.503	0.76(0.48-1.19)	0.228	0.53(0.31-0.90)	0.018	1.78(0.87-3.63)	0.113
Ischemic stroke	1.70(1.11-2.58)	0.014	2.14(1.66-2.76)	<0.001	2.72(1.85-4.00)	<0.001	2.20(1.48-3.27)	<0.001	2.53(0.99-6.45)	0.052
Hemorrhagic stroke	1.42(0.77-2.62)	0.268	1.50(1.00-2.25)	0.049	1.79(1.02-3.15)	0.044	0.73(0.23-2.30)	0.594	0.67(0.09-4.87)	0.692
Coronary artery disease	1.64(0.97-2.76)	0.064	1.58(1.19-2.08)	0.002	1.33(0.86-2.06)	0.208	1.61(1.09-2.37)	0.0163	2.28(1.01-5.12)	0.047
Peripheral artery disease	2.61(0.36-18.73)	0.340	1.76(0.94-3.29)	0.078	1.05(0.26-4.21)	0.951	0.85(0.21-3.42)	0.818	-	-
Heart failure	1.99(1.17-3.41)	0.012	2.06(1.48-2.86)	<0.001	3.64(2.36-5.61)	<0.001	4.29(2.85-6.46)	<0.001	6.00(2.94-12.24)	<0.001
Chronic kidney disease	1.46(0.92-2.30)	0.106	2.09(1.62-2.69)	<0.001	3.57(2.48-5.13)	<0.001	4.05(2.81-5.85)	<0.001	6.82(3.54-13.13)	<0.001
End-stage renal disease	1.29(0.76-2.21)	0.346	1.60(1.12-2.30)	0.011	2.19(1.16-4.16)	0.016	2.68(1.42-5.08)	0.002	3.70(1.32-10.39)	0.013
Cigarette smoking	0.82(0.56-1.20)	0.303	0.69(0.54-0.87)	0.002	0.51(0.34-0.75)	<0.001	0.47(0.32-0.69)	<0.001	0.73(0.38-1.40)	0.345
Alcohol consumption	0.57(0.37-0.87)	0.009	0.42(0.33-0.54)	<0.001	0.34(0.23-0.50)	<0.001	0.35(0.25-0.51)	<0.001	0.43(0.21-0.87)	0.020
Acute HMOD	1.61(1.15-2.25)	0.005	1.99(1.64-2.42)	<0.001	1.85(1.36-2.51)	<0.001	2.05(1.52-2.76)	<0.001	4.49(2.36-8.52)	<0.001

*The body-mass index was <18.5 in underweight group, 18.5 to 22.9 in normal group, 23.0 to 24.9 in overweight subjects, 25.0 to 29.9 in obese class I group, and ≥30.0 in obese class II + III group. BMI, body mass index; HR, hazard ratio; CI, confidence interval; HMOD, hypertension-mediated organ damage

Supplementary Table S3. Risk of 3-year all-cause mortality according to BMI categories in subgroups.

	**Adjusted hazard ratios and 95% confidence interval for 3-year all-cause mortality**				
	**Stratified by age groups**				
Aged <50 years	Underweight	Normal	Overweight	Obese class I	Obese class II+III
Unadjusted	0.89 (0.27-3.01)	Ref.	0.55 (0.23-1.29)	0.56 (0.29-1.09)	0.37 (0.15-0.92)
Model 1[†]	1.09 (0.32-3.67)	Ref.	0.46 (0.19-1.10)	0.50 (0.25-0.99)	0.39 (0.15-0.97)
Model 2[‡]	1.12 (0.33-3.82)	Ref.	0.48 (0.20-1.16)	0.57 (0.28-1.15)	0.41 (0.16-1.04)
Model 3[↑]	1.13 (0.33-3.86)	Ref.	0.50 (0.21-1.21)	0.56 (0.28-1.14)	0.41 (0.16-1.04)
Aged 50~59 years	Underweight	Normal	Overweight	Obese class I	Obese class II+III
Unadjusted	3.08 (1.60-5.96)	Ref.	0.74 (0.43-1.29)	0.41 (0.24-0.71)	0.66 (0.28-1.58)
Model 1[†]	3.30 (1.71-6.39)	Ref.	0.72 (0.42-1.26)	0.38 (0.22-0.67)	0.65 (0.27-1.56)
Model 2[‡]	3.41 (1.74-6.67)	Ref.	0.77 (0.44-1.35)	0.41 (0.23-0.74)	0.68 (0.28-1.66)
Model 3[↑]	3.33 (1.69-6.53)	Ref.	0.78 (0.45-1.37)	0.42 (0.23-0.74)	0.67 (0.28-1.64)
Aged 60~69 years	Underweight	Normal	Overweight	Obese class I	Obese class II+III
Unadjusted	2.26 (1.33-3.85)	Ref.	0.56 (0.35-0.89)	0.34 (0.21-0.55)	0.48 (0.21-1.12)
Model 1[†]	2.18 (1.28-3.72)	Ref.	0.54 (0.34-0.87)	0.34 (0.21-0.55)	0.50 (0.22-1.17)
Model 2[‡]	1.70 (0.96-3.01)	Ref.	0.52 (0.32-0.84)	0.37 (0.23-0.61)	0.49 (0.21-1.15)
Model 3[↑]	1.72 (0.97-3.03)	Ref.	0.52 (0.32-0.84)	0.37 (0.23-0.62)	0.48 (0.20-1.13)
Aged ≥70 years	Underweight	Normal	Overweight	Obese class I	Obese class II+III
Unadjusted	1.51 (1.21-1.88)	Ref.	0.59 (0.48-0.73)	0.55 (0.44-0.67)	0.52 (0.34-0.79)
Model 1[†]	1.49 (1.20-1.87)	Ref.	0.65 (0.52-0.80)	0.62 (0.50-0.77)	0.66 (0.43-0.999)
Model 2[‡]	1.45 (1.16-1.82)	Ref.	0.65 (0.52-0.80)	0.61 (0.49-0.76)	0.64 (0.41-0.98)
Model 3[↑]	1.46 (1.17-1.84)	Ref.	0.65 (0.52-0.80)	0.61 (0.49-0.76)	0.62 (0.40-0.96)
	**Stratified by presence of diabetes mellitus**				
Diabetes mellitus (no)	Underweight	Normal	Overweight	Obese class I	Obese class II+III

Unadjusted	1.92 (1.52-2.43)	Ref.	0.56 (0.45-0.71)	0.33 (0.26-0.42)	0.25 (0.16-0.40)
Model 1[†]	1.56 (1.23-1.98)	Ref.	0.62 (0.49-0.78)	0.48 (0.38-0.62)	0.64 (0.40-1.01)
Model 2[‡]	1.44 (1.13-1.84)	Ref.	0.63 (0.50-0.80)	0.49 (0.38-0.63)	0.65 (0.41-1.03)
Model 3[†]	1.44 (1.13-1.84)	Ref.	0.63 (0.50-0.79)	0.48 (0.38-0.62)	0.63 (0.39-0.998)
Diabetes mellitus (yes)	Underweight	Normal	Overweight	Obese class I	Obese class II+III
Unadjusted	2.19 (1.57-3.06)	Ref.	0.61 (0.46-0.82)	0.50 (0.38-0.65)	0.36 (0.23-0.56)
Model 1[†]	1.96 (1.40-2.74)	Ref.	0.62 (0.47-0.83)	0.56 (0.43-0.73)	0.51 (0.32-0.80)
Model 2[‡]	1.95 (1.38-2.74)	Ref.	0.64 (0.48-0.86)	0.59 (0.45-0.78)	0.56 (0.35-0.89)
Model 3[†]	1.98 (1.40-2.79)	Ref.	0.65 (0.48-0.87)	0.60 (0.46-0.78)	0.55 (0.35-0.88)
	**Patients without heart failure, ischemic stroke, hemorrhagic stroke, and end-stage renal disease**				
	Underweight	Normal	Overweight	Obese class I	Obese class II+III
Unadjusted	1.89 (1.47-2.43)	Ref.	0.52 (0.41-0.65)	0.39 (0.32-0.49)	0.27 (0.18-0.40)
Model 1[†]	1.64 (1.27-2.11)	Ref.	0.54 (0.43-0.68)	0.53 (0.42-0.66)	0.54 (0.35-0.81)
Model 2[‡]	1.56 (1.21-2.02)	Ref.	0.55 (0.44-0.69)	0.51 (0.41-0.64)	0.53 (0.35-0.80)
Model 3[†]	1.56 (1.20-2.01)	Ref.	0.55 (0.43-0.69)	0.52 (0.41-0.65)	0.53 (0.35-0.81)

*The body-mass index was <18.5 in underweight group, 18.5 to 22.9 in normal group, 23.0 to 24.9 in overweight subjects, 25.0 to 29.9 in obese class I group, and ≥30.0 in obese class II + III group. BMI, body mass index. Values are given as hazard ratio (95% confidence interval).

[†]Adjusted for age and sex.

[‡]Adjusted for age, sex, hypertension, diabetes mellitus, dyslipidemia, ischemic stroke, hemorrhagic stroke, chronic kidney disease, end-stage renal disease, smoking, and alcohol.

[†]Adjusted for age, sex, hypertension, diabetes mellitus, dyslipidemia, ischemic stroke, hemorrhagic stroke, coronary artery disease, heart failure, chronic kidney disease, end-stage renal disease, smoking, and alcohol.
